# A 3D-Printed Micro-Solid-Phase Extraction Device with Hypercrosslinked Polystyrene Sorbents for Highly Reproducible Aromatic Acid Analysis in Blood Serum

**DOI:** 10.3390/ijms27146443

**Published:** 2026-07-20

**Authors:** Alisa K. Pautova, Alexander Y. Popov, Andrey S. Samokhin, Natalia V. Beloborodova, Alexander I. Revelsky

**Affiliations:** 1 Federal Research and Clinical Center of Intensive Care Medicine and Rehabilitology, Petrovka str., 25-2, 107031 Moscow, Russia; nbeloborodova@fnkcrr.ru; 2 Efferon JSC, Leninskie Gory 1/75G, 119234 Moscow, Russia; a.popov@efferon.ru; 3 Chemistry Department, Lomonosov Moscow State University, GSP-1, Leninskie Gory, 1-3, 119991 Moscow, Russia; andrey.s.samokhin@gmail.com (A.S.S.); sorbent@yandex.ru (A.I.R.)

**Keywords:** phenyl-containing acids, microextraction by packed sorbent, microextraction in packed syringe, 3D printing, gas chromatography–mass spectrometry

## Abstract

Modern methods for preparing biological samples prior to chromatography–mass spectrometry analysis are rapidly developing and include both commercial platforms and in-house devices designed for specific sample-preparation conditions. Here, we demonstrate the feasibility of using hypercrosslinked polystyrene, packed into disposable plastic pipette tips, as a sorbent for micro-solid-phase extraction. This approach was combined with a custom 3D-printed device designed to perform sample preparation similar to that of commercial microextraction by packed sorbent (MEPS) systems. Extraction conditions were developed for aromatic metabolites of tyrosine and phenylalanine in human blood serum, followed by gas chromatography–mass spectrometry analysis of their silyl derivatives. The proposed method was evaluated using serum samples from healthy donors. Two types of hypercrosslinked polystyrene with nominal particle sizes of 40 and 60 µm were used. Under the optimized conditions, the mean normalized recoveries reached 90% for benzoic acid and 60–80% for most analytes, whereas phenyllactic and 4-hydroxyphenyllactic acids, the two most polar analytes, remained at lower recoveries. Moreover, the 3D-printed device achieved reproducibility comparable to that of a commercial MEPS system, with relative standard deviations below 10% under optimized conditions. The proposed in-house device also offers a fast and efficient strategy for evaluating custom sorbents in various sample-preparation applications.

## 1. Introduction

Analytical techniques with high selectivity and sensitivity, such as gas chromatography coupled with mass spectrometry (GC-MS), are widely applied to analyze biological fluids and gain insights into the complex composition of low-molecular-weight organic metabolites [1]. A sample preparation step is often required to separate target analytes from matrix compounds prior to GC-MS analysis [2]. Solid-phase extraction (SPE), and especially its miniaturized variant, microextraction by packed sorbent (MEPS), has been actively studied to address various practical bioanalytical issues [3,4,5,6,7].

Commercial MEPS systems enable sample preparation from microliter-scale sample volumes, adjustment of the number of aspiration/dispensing cycles, precise control of flow rates, low consumption of organic eluent, repeated use of the barrel-insert needle after appropriate washing, and straightforward automation with online coupling to chromatographic systems. These advantages explain the rapid integration of MEPS technology into analytical practice after its commercialization by SGE Analytical Science, as well as the subsequent expansion of its application areas [8]. Nevertheless, several factors still restrict the wider use of MEPS systems in routine and research laboratories. The most important limitations are the narrow range of commercially available sorbents and the high cost of both the hardware and replaceable needle assemblies. Consequently, considerable effort has been directed toward developing low-cost MEPS-like alternatives based on in-house cartridges containing only milligram amounts of sorbent, as well as devices that enable semi- or fully automated workflows.

Several strategies for packing small amounts of sorbent have been reported in the literature. One of the simplest and most easily reproducible approaches involves placing the sorbent near the outlet of a 1.0 mL polypropylene insulin syringe between two frits [9,10,11,12]. Other technically advanced solutions more closely mimic commercial MEPS systems, as they use a laboratory-made extraction cartridge positioned between a removable needle and a glass syringe [13,14,15]. This design is attractive for analytical laboratories because the sorbent cartridge can be removed, repacked, and reused. However, the fabrication of small polytetrafluoroethylene components is not readily accessible to all laboratories because it requires precision micro-machining. More accessible strategies rely on disposable plastic pipette tips, as used in pipette-tip micro-SPE [16] and disposable pipette extraction [17]. In addition to conventional packed-bed formats, some studies have introduced in situ polymerization inside plastic tips to obtain monolithic stationary phases [18,19,20].

Automation is another important component of the MEPS workflow because it improves reproducibility through precise control of flow rates and aliquot volumes, reduces operator-dependent steps, and increases the overall productivity of analytical laboratories. Early studies from the 2010s addressed this aspect by using simple DC motor-based actuators to automate aspiration/dispensing steps [9,12]. More recent studies have described Cartesian robots controlled by Arduino boards [13,21], enabling not only accurate control of plunger movement but also the automation of multistep sample preparation workflows. In these systems, task-specific components, such as syringe holders and plunger-driving mechanisms, were fabricated from acrylic, most likely by milling or related subtractive manufacturing techniques. However, the rapid development of 3D printing technologies has made the fabrication of custom components for in-house analytical devices more accessible and cost-effective. Accordingly, 3D printing has already been used to create various laboratory tools and analytical devices, including micropipettes [22], orbital shakers [23], syringe pumps [24], peristaltic pumps [25], and autosamplers [26]. In this context, 3D printing offers an attractive strategy for developing low-cost MEPS-like devices that meet the requirements of modern analytical laboratories.

Various modifications of hypercrosslinked polystyrene (HCLPS) developed by the group of Prof. Davankov V.A. and Tsyurupa M.P. are of scientific interest. During synthesis, HCLPS can be tailored in terms of porous structure and functionalization. In contrast to conventional styrene-divinylbenzene copolymers, HCLPS is obtained by crosslinking dissolved or highly swollen polystyrene chains with spacer bridges rather than by monomer copolymerization. This process yields a highly crosslinked, single-phase polymer network with characteristic properties arising at a crosslinking degree of at least 40%, whereas typical HCLPS materials have crosslinking degrees of 100–200% (defined as the average number of cross-bridges per phenyl ring, expressed as a percentage). This highly crosslinked structure can be represented as a large number of intercondensed and interpenetrating macrocycles [27]. Depending on its structural characteristics, HCLPS may exhibit various properties suited to a broad range of applications, including the extraction of organic molecules [28,29] and heavy metals [30] from complex matrices, gas separation [31], and blood purification in clinical medicine [32,33,34].

The MEPS technique has been used in our laboratory to determine aromatic metabolites of tyrosine and phenylalanine, i.e., phenyl-containing acids, which are considered to be markers of infectious processes in critically ill patients [35]. Initially, a commercially available MEPS device with C18 barrel-insert needles was used to extract these compounds from blood serum at micromolar concentrations [36]. This approach provided reproducibility and sensitivity comparable to those of traditional liquid–liquid extraction, while reducing the required sample volume from 200 to 80 μL [37]. However, after analyzing serum samples from patients with sepsis, the commercial C18 sorbent-containing needles were found to be non-reusable. This limitation encouraged us to use tailor-made HCLPS as an alternative sorbent that could be repeatedly regenerated. Subsequently, HCLPS was evaluated for the analysis of phenyl-containing acids using both traditional SPE and its in-house miniaturized variant with 5 mg of sorbent [38]. Since all operations were performed manually, the miniaturized approach exhibited lower reproducibility, with relative standard deviation (RSD) values of 10–20%, compared with the previously tested commercial MEPS system, which had RSD values of 5–10% [36]. At the same time, both approaches shared a common limitation: low analyte-normalized recoveries, ranging from 20% to 80%. Thus, further development should focus on improving analyte recoveries to enhance method sensitivity and reproducibility when using custom sorbents.

The goal of the current study was to design and manufacture a custom in-house device with functionality comparable to commercial MEPS systems. Additionally, we aimed to develop a simple, laboratory-assembled alternative to conventional sorbent-packed MEPS needles, and to demonstrate its feasibility for extracting phenyl-containing acids, as model compounds of varying polarities, from blood serum samples. Since the analytical performance of sorbents is influenced by their pore structure and particle diameter, two types of HCLPS with nominal particle sizes of 40 and 60 µm were evaluated. Although 3D-printed syringe-pump-based devices have been described previously, this work focuses on their practical implementation in a MEPS-like sample-preparation format. In line with the goal of creating a reproducible in-house alternative to commercial MEPS systems, we prioritized accessible, low-cost fabrication solutions. Therefore, custom-designed components of the device were manufactured using 3D printing, and disposable plastic tips were used as sorbent containers.

## 2. Results and Discussion

### 2.1. Hypercrosslinked Polystyrene Properties

HCLPS exhibits broad applicability as a sorbent due to strong hydrophobic and π-π interactions between the sorbent polymer network and various analyte molecules. Depending on the synthesis route and starting polymer, HCLPS materials can differ substantially in pore architecture, particle morphology, swelling behavior, and accessibility to the matrix component. These properties are particularly important in MEPS and pipette-tip SPE, where small amounts of sorbent are used, and the sample is repeatedly passed through the sorbent bed. MEPS is generally implemented in a syringe-based device, whereas in-tip SPE uses a sorbent packed directly into a disposable pipette tip; both formats follow the same packed-sorbent microextraction principle. Restricted access material/media (RAM) sorbents are attractive for such applications because they retain low-molecular-weight analytes while excluding proteins and other macromolecular matrix components, potentially reducing matrix effects, fouling, and carryover [39,40,41]. However, direct comparisons of RAM and non-RAM sorbents under identical MEPS-like conditions remain limited. Therefore, in this study, two HCLPS sorbents with nominal particle sizes of 40 and 60 µm were evaluated: one was synthesized in-house (D2H2), whereas the other (MN200) was commercially available (Table 1).

Microporous D2H2 was selected as a RAM-type sorbent because materials of this type retain low-molecular-weight organic compounds while excluding albumin and other proteins. Specifically, under static testing conditions (0.125 g of sorbent in contact with 10 mL of a 0.1% protein solution in phosphate buffer at pH 6.86 for 3 h), D2H2 demonstrated < 0.1% adsorption of both human serum albumin and cytochrome C [42]. Unlike the microporous RAM-type sorbent, MN200 contains transport pores and retains protein components; under identical static conditions, it adsorbed 89% of human serum albumin and 99% of cytochrome C [42,43]. Because the original MN200 resin is supplied as relatively large spherical beads, with a typical particle-size range of 300–1200 µm, it cannot be directly packed into pipette tips used for the MEPS technique. Therefore, MN200 was mechanically ground, and the 40–63 µm particle-size fraction was collected before use. Thus, the study included two practically different HCLPS-based sorbents: spherical microporous RAM-type particles and irregularly shaped particles of ground biporous MN200.

### 2.2. Extraction of Phenyl-Containing Acids

HCLPS was obtained in a particulate form, and was dispersed in a 20% aqueous acetone solution to prepare a suspension containing 3 mg of sorbent per 30 µL. To assemble the extraction device, a 1 mm-thick hydrophilized polyethylene limiter was placed in the narrow section of a 200 µL pipette tip, followed by the addition of 30 µL of the HCLPS suspension. The solid particles settled onto the lower limiter while the excess solvent drained by gravity. Finally, a second limiter was inserted to secure the sorbent bed, and the assembled tips were stored wet to prevent excessive drying of the sorbent before use.

**Figure 1:**
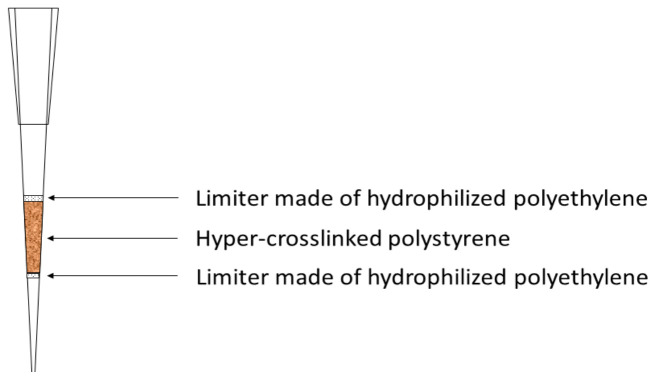
The schematic of the tip with HCLPS sorbent.

A schematic representation of the assembled tip with HCLPS sorbent is shown in Figure 1.

**Figure 2:**
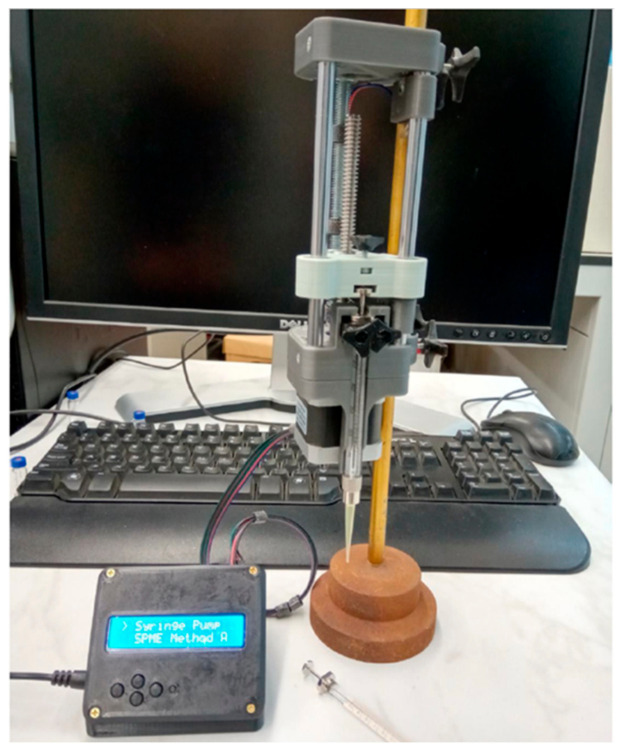
A general view of the 3D-printed MEPS-like device with a pipette tip packed with HCLPS sorbent.

The extraction of the phenyl-containing analytes was performed using an in-house 3D-printed MEPS-like device (see Section 3.3). In the present study, only a single device was fabricated; therefore, inter-device reproducibility was not evaluated directly. Nevertheless, a closely related hardware design was previously used to dispense small aliquots of organic solvents onto the surface of photonic-crystal sensors [44]. Both devices demonstrated low dispensing errors when operated with 50–500 µL glass microsyringes. Dispensing accuracy and reproducibility ranged from 0.05 to 0.3 µL depending on the aliquot volume and syringe size. These results were obtained after gravimetric calibration, which was required to minimize systematic errors associated with individual device components. A syringe was mounted inside the device, and plunger movement was controlled according to operator-defined parameters, including volume, flow rate, and number of sequential operations (Figure 2). The use of HCLPS-packed pipette tips instead of barrel-insert needles allowed the extraction to be performed without direct contact between the sample and the syringe, reducing syringe contamination and the risk of cross-contamination.

**Figure 3:**
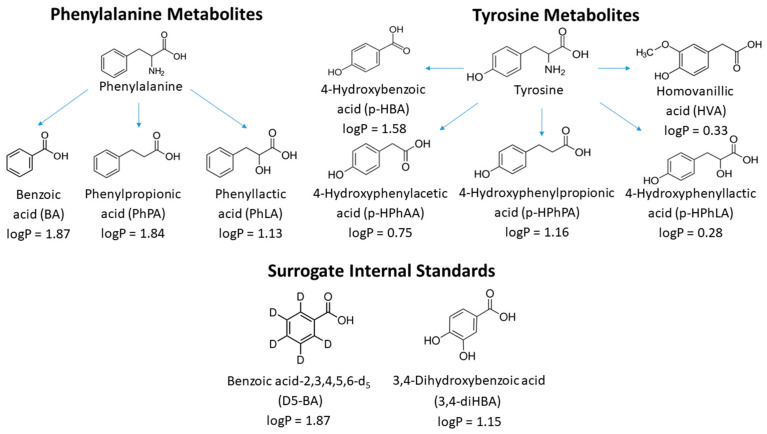
Structures and logP values of phenyl-containing metabolites of phenylalanine and tyrosine, and surrogate internal standards.

Model compounds of varying polarity were examined, including phenyl-containing metabolites of phenylalanine (benzoic (BA), phenylpropionic (PhPA), and phenyllactic (PhLA) acids) and tyrosine (4-hydroxybenzoic (p-HBA), 4-hydroxyphenylacetic (p-HPhAA), 4-hydroxyphenylpropionic (p-HPhPA), homovanillic (HVA), and 4-hydroxyphenyllactic (p-HPhLA) acids), along with two surrogate internal standards (benzoic acid-2,3,4,5,6-d_5_ (D5-BA) and 3,4-dihydroxybenzoic acid (3,4-diHBA)) (Figure 3).

The analytical characteristics of both microporous RAM and biporous HCLPS sorbents were evaluated by extracting phenyl-containing acids from both diluted and undiluted blood serum samples of healthy donors after spiking with model compounds. The experimental conditions were adapted from our previous work [38], in which all operations with the in-house HCLPS-packed plastic tip were performed manually. Briefly, the workflow included sorbent conditioning, repeated aspiration/dispensing of serum through the packed tip, washing and drying of the sorbent, acetone desorption, and regeneration of the sorbent between sample series. The specific solvent volumes, cycle numbers, and flow rates are provided in Section 3.5. After desorption, the eluate was evaporated to dryness, derivatized with N,O-bis(trimethylsilyl)trifluoroacetamide (BSTFA), and dissolved in hexane containing hexachlorobenzene prior to GC-MS analysis. The mean normalized recoveries (MNR) of the analytes and RSD values obtained after extraction from serum samples using both sorbents are summarized in Table 2.

To evaluate the effects of experimental conditions on MNR and reproducibility, we applied a generalized linear mixed model (GLMM) rather than treating measurements obtained from individual chromatographic runs as independent observations. Since all target acids were extracted from the same serum samples using common MEPS conditions, observed responses reflected both analyte-specific extraction behavior and sample-level variability shared across analytes. Mixed-effects modeling is appropriate for such analytical datasets, since it allows structured variability to be incorporated into the model instead of being pooled into a single residual error term. Similar logic has been used in analytical method evaluation to account for sample-, analyst-, and batch-related variability [45], and in analytical method optimization using a factorial GC–MS/MS design, with experimental block as a random effect [46].

In the present study, this strategy was implemented using the glmmTMB package [47] by including analyte and analytical-run identifiers as random effects and by modeling both normalized recovery and residual variance (the raw model console output is provided in Supplementary Section S1, including Figures S1–S3). By estimating all prespecified effects within a single hierarchical model, rather than performing separate analyte-wise tests, this approach reduces the multiplicity problem and provides conservative inference for the limited number of independent matrix replicates (n = 3). This was important, since higher extraction efficiency is analytically useful only when accompanied by acceptable reproducibility. The model provided two separate types of effect ratios. Ratios from the MNR component describe how the experimental factors changed the expected normalized recovery, with values above 1 indicating higher normalized recovery. Ratios from the variance component describe how the same factors changed the residual relative variability of the normalized recoveries; values below 1 indicate a more stable extraction process, consistent with improved reproducibility and lower RSD values. To make the interpretation of the model outputs immediately accessible, a graphical summary of the key predictions (marginal effects) is presented in Supplementary Figure S1 in Supplementary Section S1.

In our previous liquid–liquid extraction studies, several surrogate internal standards were used to improve reproducibility [37]. These compounds were selected to resemble the structural and chemical properties of the respective analytes. D5-BA was used for BA and PhPA, both of which contain a single polar carboxyl group. The other hydroxy acids are more polar because they contain one or two additional hydroxy groups; for these analytes, better reproducibility was achieved using more polar surrogate internal standards, such as 3,4-diHBA and D3-p-HPhLA. However, under MEPS conditions, only D5-BA showed reasonable and reproducible MNR. These results were previously reported [36,38] and were reproduced in the current study. Therefore, the more polar surrogate internal standards were excluded from further consideration, and D5-BA was used to normalize the responses of all target analytes. Under the selected MEPS conditions, only BA (and its deuterated standard) showed MNR close to 100% (Table 2). More polar phenyl-containing acids with a phenolic hydroxy group exhibited lower MNR, ranging from 51 to 70%, in agreement with the logP effect estimated by the GLMM (ratio = 1.54). At the same time, analytes with a hydroxy group adjacent to the carboxyl group showed the lowest MNR, ranging from 19 to 30%.

According to the GLMM, the HCLPS sorbent type did not significantly affect MNR (*p* = 0.323). However, the dispersion component revealed a significant interaction between sorbent type and sample dilution (ratio = 1.42, [95% CI: 1.07–1.88]). In undiluted samples, D2H2, a sorbent with RAM properties, showed lower RSD values (≤8%, except for p-HPhLA), whereas MN200 exhibited greater variability (RSD values of 13–28% for half of the analytes). Conversely, in twofold-diluted serum, the biporous MN200 achieved a highly significant reduction in variance over D2H2 (ratio = 0.65, [95% CI: 0.53–0.80]). These results suggest that while RAM-type D2H2 was more effective in undiluted matrices, biporous MN200 showed better reproducibility after matrix dilution, possibly due to the differences in pore structure and morphology. Sorbent fouling was suggested by reduced sample passage through the sorbent during the aspiration stage and a noticeable decrease in the chromatographic peak areas of the analyte derivatives. The experiments showed that tips packed with D2H2 or MN200 could be used to analyze up to 12 serum samples without replacement.

We encountered several challenges when working with undiluted serum samples. First, repeated aspiration/dispensing cycles resulted in a thin foam layer (approximately 1 mm thick) above the packed sorbent. The foamed portion of the sample (about 10–15 µL) could not be completely removed from the tip during dispensing, most likely because of surface-tension effects. Second, after sorbent drying, a translucent film appeared on the inner walls of the tip, which was likely associated with protein deposition. Subsequent washing with water and acetone partially removed these deposits. Finally, attaching a plastic tip to the syringe altered the system’s behavior. Although the syringe is a positive-displacement device, the presence of an air gap between the liquid sample and the syringe plunger caused the system to behave like an air-displacement device. In addition, the packed sorbent in the tip increased flow resistance. As a result, a delay was observed between syringe plunger movement and actual filling of the tip with undiluted serum. To address these challenges, blood serum samples were diluted twofold to reduce their viscosity. This adjustment helped overcome the main problems because the delay between the syringe plunger movement and solution movement in the polypropylene tip was significantly reduced. To preserve the same amount of biological material, the aliquot volume was increased in proportion to the dilution (from 50 to 100 µL).

The MNR obtained from diluted serum samples is also summarized in Table 2. Comparing analyte MNR from diluted and undiluted samples, PhPA and PhLA demonstrated better MNR in the case of diluted serum samples for both sorbents, with similar MNR for the rest of the analytes. The GLMM confirmed that twofold dilution of the serum samples significantly increased the MNR (ratio = 1.14 [95% CI: 1.04–1.25]). Furthermore, sample dilution was a statistically significant factor in stabilizing the process and reducing variance (ratio = 0.84 [95% CI: 0.78–0.91]).

Optimization of the MEPS conditions focused mainly on the number of aspiration/dispensing cycles (4–15) during the sorption stage. According to the model, this parameter did not significantly affect MNR (*p* = 0.958). While the dispersion submodel indicated that the overall trend in reproducibility across the tested cycle range was not statistically significant (ratio = 0.84 [95% CI: 0.59–1.19]), the lowest observed RSD (RSD ≤ 10%) was achieved with seven cycles. This reproducibility, achieved with the developed device and tailor-made HCLPS sorbents, was comparable to that previously reported for a commercial MEPS system with a C18 sorbent (RSD = 5–10%) [36] and better than that obtained for a manually operated HCLPS-based approach (RSD = 10–20%) [38]. Compared with manual handling, the in-house 3D-printed MEPS-like device improved operational convenience because it could be fixed to a laboratory stand and operated via the control panel to perform a predefined sequence of aspiration/dispensing cycles. Under optimal conditions, combining the laboratory-assembled HCLPS-packed tips with a twofold dilution of serum samples significantly improved MNR, which increased to 90% for BA, 80% for PhPA, 70% for p-HBA, p-HPhPA, and HVA, and 60% for p-HPhAA. These results demonstrate that tailor-made sorbents combined with custom automation devices can improve the analytical performance of existing sample-preparation methods.

This study was intended to demonstrate the practical feasibility of the in-house MEPS-like system for blood serum sample preparation, rather than to perform comprehensive sorbent screening. For this reason, only two HCLPS sorbents were evaluated, both of which demonstrated acceptable MNR and reproducibility for the target phenyl-containing acids. These results indicate that the proposed approach can be adapted to ongoing analytical tasks in clinical and bioanalytical laboratories. Although several parameters may influence MEPS performance (including conditioning, washing, and desorption), this study focused primarily on the sorption stage as a key step affecting analytical performance. Matrix effects in serum were addressed only indirectly through dilution experiments; however, more explicit evaluation (e.g., matrix factor calculation or post-extraction spike experiments) would improve confidence in the quantitative reliability of the method in future studies. Moreover, a direct side-by-side comparison with commercial MEPS cartridges under identical extraction conditions was not performed and represents a limitation of the present study. Also, the determination of endogenous analytes in real samples was beyond the scope of this work. Instead, phenyl-containing acids with different polarities were used as model compounds to evaluate the proposed in-house MEPS-like system.

## 3. Materials and Methods

### 3.1. Reagents and Materials

Benzoic acid (BA, ≥99.5%), 2,3,4,5,6-D5-benzoic acid (D5-BA, ≥99 atom % D, ≥99%, internal standard), phenylpropionic acid (PhPA, ≥99%), phenyllactic acid (PhLA, ≥98%), 4-hydroxybenzoic acid (p-HBA, ≥99%), 4-hydroxyphenylacetic acid (p-HPhAA, ≥98%), 4-hydroxyphenylpropionic acid (p-HPhPA, ≥98%), homovanillic acid (HVA, ≥97%), 4-hydroxyphenyllactic acid (p-HPhLA, ≥97%), 3,4-dihydroxybenzoic acid (3,4-diHBA, internal standard), N,O-bis(trimethylsilyl)trifluoroacetamide (BSTFA, 99%, contains 1% trimethylchlorosilane), and formic acid (≥95%) were obtained from Merck (Darmstadt, Germany); sulfuric acid and acetone were Laboratory Reagent grade (Khimreactiv, Staryy Oskol, Russia).

Serum samples from three healthy donors were collected at the Federal Research and Clinical Center of Intensive Care Medicine and Rehabilitology (Moscow, Russia). Approval from the local Ethics Committee of the Federal Research and Clinical Center of Intensive Care Medicine and Rehabilitology was obtained (protocol code 6/25/2, 19 November 2025).

### 3.2. Preparation of Hypercrosslinked Sorbents

D2H2 was synthesized based on the previously described procedure [42], with a modified stirring rate during copolymer preparation. Briefly, a nonporous gel-type styrene–divinylbenzene copolymer containing 2 mol% divinylbenzene was obtained by suspension radical polymerization with benzoyl peroxide at 2000 rpm. The dried copolymer beads were swollen in 1,2-dichloroethane and then hypercrosslinked with monochlorodimethyl ether in the presence of FeCl_3_ to a crosslinking degree of 200%.

MN200 was a commercial HCLPS (Ecolab Purolite, Llantrisant, UK), which was mechanically ground in an agate mortar in the presence of water to improve dispersion. The 40–63 µm particle size fraction was isolated by sedimentation in acetone.

### 3.3. 3D-Printed Microextraction by Packed Sorbent-like Device

**Figure 4:**
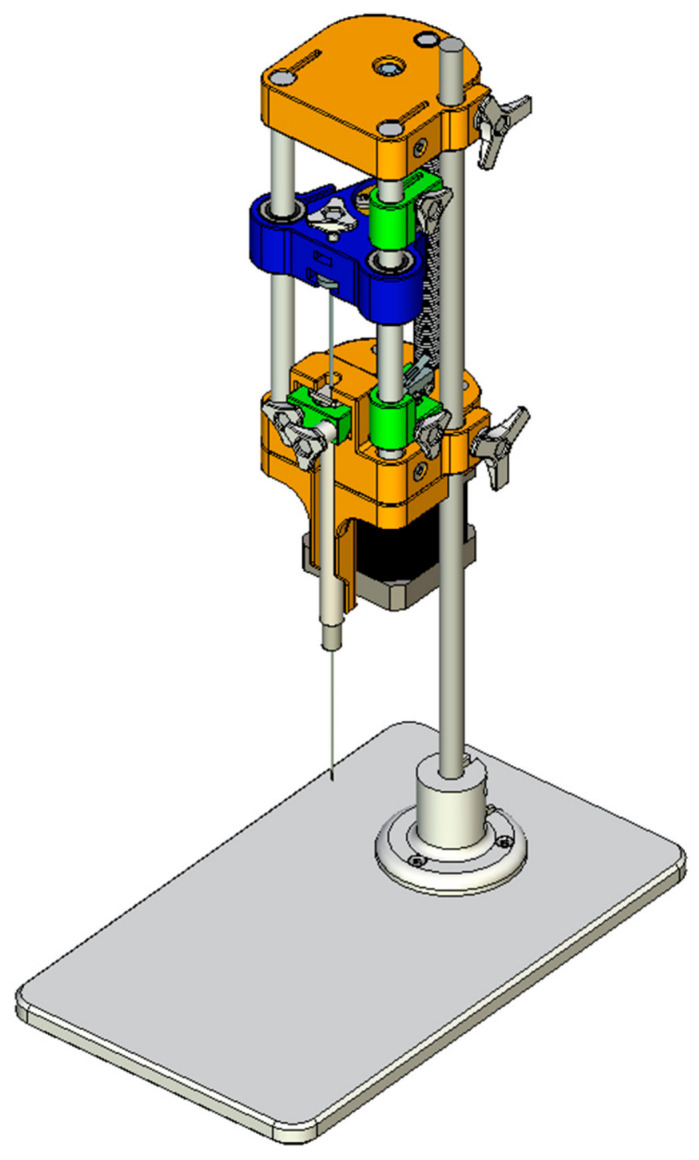
A general view of the 3D-printed MEPS-like device.

The open-source syringe pump that we designed several years ago [48] served as the basis for a 3D-printed MEPS-like device. The original design was modified to adapt it for this specific application. In particular, the syringe pump was reoriented vertically, and mounts were added to allow attachment to a standard laboratory stand. In addition, to reduce the device’s overall size, both the stepper motor and the syringe were positioned at the lower end of the lead screw. A general view of the in-house MEPS-like device is shown in Figure 4.

Both glass and disposable plastic syringes with outer diameters below 8.0 mm and standard-size flanges can be installed in the device. The main components were fabricated from polylactic acid using a custom fused deposition modeling 3D printer. The parts were designed using T-FLEX CAD 17 software (Top Systems, Moscow, Russia). The remaining components were purchased from a local hardware store, including a T8 lead screw, a T8 nut, 8 mm guide rods, LM8SUU linear bearings, a 688ZZ ball bearing, an M8 threaded rod, a NEMA17 stepper motor, and other standard parts. The design of the control panel was retained from the original syringe pump. The control panel consisted of an Arduino UNO board, an LCD Keypad Shield, an A4988 stepper motor driver, and an active buzzer. The enclosure was also 3D-printed from polylactic acid.

The original firmware was extensively modified. The main modification was the implementation of a dedicated MEPS mode. Several MEPS methods are available to the operator and can be saved in non-volatile memory. Each method includes up to five stages, and each stage is defined by the number of pumping cycles, aspiration volume, aspiration flow rate, and dispensing flow rate. Extraction from the solution is performed in semi-automatic mode. At the beginning of each aspiration or dispensing stage, the operator is notified by an audible signal. The operator then places the plastic tip into either the sample vial or waste vial, depending on the purpose of the step, and then presses a button on the control panel to continue the process.

All syringes used in this work were calibrated gravimetrically in accordance with ISO 8655 [49]. Both dispensing accuracy and reproducibility were evaluated. When a 250 µL glass syringe was used to dispense a 200 µL aliquot, both the accuracy and reproducibility were better than 0.2 µL, as determined from 10 replicate measurements.

### 3.4. Preparation of Stock and Working Solutions

#### 3.4.1. Acetone Stock Solutions

Stock solutions of analytes (BA, PhPA, PhLA, p-HBA, p-PhAA, p-PhPPA, HVA, and p-HPhLA) and the internal standards (D5-BA and 3,4-diHBA), each with a concentration of 0.0067 g/mL, were prepared by dissolving 0.01 g of pure solid standards in 1.5 mL of acetone.

#### 3.4.2. Acetone Working Solution

Aliquots (18 µL) of stock solutions of each analyte and internal standards were diluted with 1000 µL of acetone to obtain a concentration of 1.2 × 10^−7^ g/µL. An aliquot (10 µL) of the acetone working solution was diluted with 200 µL of acetone to obtain a final concentration of 5.7 × 10^−9^ g/µL. Then, 50 µL of this solution was evaporated to dryness and derivatized with 20 µL of BSTFA (30 min, 80 °C). Then, the silyl derivatives were cooled at 4 °C for 1 h and dissolved in 400 µL of a hexane solution of hexachlorobenzene.

#### 3.4.3. Serum Sample Solutions

Aliquots (18 µL) of stock solutions of each analyte and internal standards or only internal standards (in the case of blank serum sample analyses) were diluted with 1000 µL of distilled water to obtain a concentration of 1.2 × 10^−7^ g/µL. An aliquot (10 µL) of the aqueous working solution was added to 200 µL of pooled serum, prepared from the serum samples of three healthy donors, to obtain a concentration of 5.7 × 10^−9^ g/µL. Twofold-diluted serum samples were prepared using a 0.2% formic acid solution. An aliquot (50 µL for undiluted or 100 µL for diluted serum samples) of this solution was used for further microextraction sample preparation using two HCLPS sorbents.

### 3.5. Microextraction from Diluted Serum Samples

- Sample volume: 100 µL (50 µL for undiluted serum samples);
- Sorbent conditioning: acetone 6 × 100 µL (50 µL for undiluted serum samples), water, 1% formic acid solution 3 × 100 µL (50 µL for undiluted serum samples), flow rate—15 µL/s for each solvent;
- Sorption: diluted serum samples, 4/7/10/15 × 100 µL (50 µL for undiluted serum samples), flow rate—5 µL/s;
- Sorbent washing: 0.1% formic acid solution, 2 × 20 µL, flow rate: 8 µL/s;
- Sorbent drying: air, 5 × 50 µL, flow rate: 15 µL/s;
- Desorption: acetone, 10 × 20 µL, flow rate—10 µL/s;
- Evaporation: to dryness;
- Derivatization: BSTFA, 20 µL, 30 min, 80 °C;
- Sorbent regeneration between sample series: 0.9% NaCl solution, distilled water, and acetone (4 × 100 μL for each solvent), followed by distilled water and 1% formic acid solution (3 × 50 μL); flow rate—15 µL/s for each solvent.

After derivatization, the mixture was cooled at 4 °C for 1 h and dissolved in a 400 µL aliquot of hexane containing hexachlorobenzene prior to GC-MS analysis.

### 3.6. GC-MS Conditions

GC-MS analysis was conducted on a Trace GC 1310 ISQ LT Thermo Scientific instrument (Thermo Electron Corporation, Waltham, MA, USA), equipped with an AI 1310 automatic liquid sampler (Thermo Electron Corporation, Waltham, MA, USA). Separation was carried out on a TR-5ms quartz capillary column (Thermo Electron Corporation, Waltham, MA, USA): stationary phase 95% dimethylpolysiloxane, 5% phenylpolysiloxane, length 30 m, inner diameter 0.25 mm, and stationary phase thickness 0.25 µm. The chromatographic separation conditions were as follows: injector temperature 200 °C; carrier gas (helium) flow rate 1.5 mL/min; and split-flow mode (1:5). The volume of the injected sample was 1 µL. The column oven temperature program was as follows: initial temperature of 80 °C, held for 4 min; ramped to 250 °C at 10 °C/min; then ramped to 280 °C at 20 °C/min; and held at 280 °C for 5 min. The total analysis time was 27.5 min. The mass spectrometric analysis conditions were as follows: electron ionization (70 eV), interface temperature 250 °C, ionization chamber temperature 200 °C, *m*/*z* scanning range 50–450 amu, scanning rate 3 scans per second, and the cathode was turned on 2 min after the start of the analysis.

D5-BA was used as a surrogate internal standard for BA and PhPA; 3,4-diHBA was tested as a surrogate internal standard for PhLA, p-HBA, p-PhAA, p-PhPPA, HVA, and p-HPhLA; however, all subsequent quantifications were performed using D5-BA and hexachlorobenzene was used as an instrumental internal standard. The *m*/*z* values for quantification were selected based on their informativeness (reflecting the compound’s structure) and their absence in the background mass spectrum. Their values, expected fragmentation, retention times, and retention indices have been described previously [37] and are briefly summarized in Supplementary Table S1. Representative chromatograms generated by summing the extracted-ion traces for all selected quantification ions are presented in Supplementary Figures S4 and S5 for pooled blood serum from healthy donors spiked with internal standards and analytes or with internal standards only, respectively.

The normalized recoveries of the analytes were calculated as follows:

$$Normalized\;Recovery,\;\%=\;\frac{\frac{Analyte\;Area\;in\;Serum\;Solution}{Internal\;Standard\;Area\;in\;Serum\;Solution}-\frac{Analyte\;Area\;in\;Blank\;Serum}{Internal\;Standard\;Area\;in\;Blank\;Serum}}{\frac{Analyte\;Area\;in\;Acetone\;Solution}{Internal\;Standard\;Area\;in\;Acetone\;Solution}}\times 100$$

where *Analyte Area in Serum Solution*—the peak area of the silyl derivative of the analyte in the serum solution with added analytes after sample preparation;

*Internal Standard Area in Serum Solution*—the peak area of the silyl derivative of the surrogate internal standard in the serum solution with added analytes after sample preparation;

*Analyte Area in Blank Serum*—the peak area of the silyl derivative of the analyte in the blank serum without added analytes after sample preparation;

*Internal Standard Area in Blank Serum*—the peak area of the silyl derivative of the surrogate internal standard in the blank serum without added analytes after sample preparation;

*Analyte Area in Acetone Solution*—the peak area of the silyl derivative of the analyte in acetone solution without sample preparation (only derivatization);

*Internal Standard Area in Acetone Solution*—the peak area of the silyl derivative of the surrogate internal standard in acetone solution without sample preparation (only derivatization).

The mean normalized recovery (MNR, %) and relative standard deviation (RSD, %) were calculated based on the results of three replicate experiments using Microsoft Excel (*p* = 0.95). All raw data on relative signals, MNR, and RSD are compiled in Supplementary Table S2.

### 3.7. Statistical Analysis

Statistical analysis was performed in R using the glmmTMB package [47]. To simultaneously evaluate factors affecting both the extraction efficiency and method reproducibility, a GLMM was applied to the log-transformed, normalized recovery data with a Gaussian distribution. Sorbent type, number of cycles (as 1/n), sample dilution, and log P were defined as fixed effects for both the conditional (mean normalized recovery) and dispersion (residual variance/RSD) components of the model. Additionally, a sorbent-by-dilution interaction term was included in the variance component to account for matrix-dependent changes in reproducibility. To account for the hierarchical structure of the dataset, analyte and analytical-run identifier were included as random intercept effects in the mean-recovery component. Each analytical-run identifier corresponds to a single GC-MS run; responses for all analytes measured during that run were grouped together. Coefficients from the mean recovery component were back-transformed to recovery ratios as exp(β), representing fold-changes in expected normalized recovery. Coefficients from the variance component were back-transformed as exp(β/2) and interpreted as relative-SD-scale ratios. Since the response was log-transformed, these ratios reflect model-estimated changes in residual relative variability rather than exact empirical RSD ratios; values below 1 indicate improved reproducibility. All data for this analysis are compiled in Supplementary Section S1.

The lipophilicity (logP) values used for the target analytes were obtained via the SwissADME platform, utilizing consensus experimental data where available, and XLOGP3 algorithmic predictions for the remaining compounds.

## 4. Conclusions

This study demonstrates that a MEPS-like sample-preparation device can be developed in-house using readily accessible components and 3D-printed parts. The proposed device offers a low-cost alternative to commercial MEPS systems, providing comparable functionality while avoiding reliance on proprietary hardware and sorbent-packed needles. Importantly, the device can be used with tailor-made sorbent cartridges, enabling tuning of extraction performance at the sorbent level rather than relying solely on commercially available stationary phases. The choice of HCLPS sorbent had little effect on analyte recovery but strongly influenced reproducibility through pore architecture and sample preparation. RAM-type pores improved reproducibility in undiluted serum, whereas the biporous structure provided better reproducibility after sample dilution. Therefore, the developed solution represents an accessible alternative to commercial MEPS systems and may serve as a convenient tool for evaluating selected sorbent materials under defined specific analytical conditions. Its successful application to the extraction of phenyl-containing acids from blood serum demonstrates the feasibility of this approach for sample preparation in complex biological matrices.

## Figures and Tables

**Table 1:** Characteristics of the evaluated hypercrosslinked polystyrene (HCLPS) sorbents.

Characteristics	D2H2	MN200
Origin and sorbent type	Laboratory-synthesized, microporous RAM ^1^-type HCLPS	Commercial, biporous HCLPS
Nominal crosslinking degree	200%	200%
Median (P90) particle diameter in swollen state/dry state, µm	38 (30–45)/ 34 (26–41)	56 (40–63)/ 55 (39–62)
Pore diameter, nm	0.7–2	0.9–5, 60–110
Specific surface area by BET ^2^, m^2^/g	914	932
Water regain (total pore volume in water), mL/g	0.71	1.55
Toluene regain, mL/g	0.97	1.63
Wetting behavior	Requires organic prewetting	Directly water-wettable

^1^ RAM—restricted-access material/media and ^2^ BET—Brunauer–Emmett–Teller technique.

**Table 2:** Mean normalized recoveries (MNR, %) and relative standard deviations (RSD, %) for phenyl-containing acids extracted from undiluted and diluted serum samples using the in-house microextraction by packed sorbent (MEPS)-like device with hypercrosslinked polystyrene (HCLPS) sorbents D2H2 and MN200. The number of aspiration/dispensing cycles during the sorption stage with MN200 ranged from 4 to 15.

Sorbent	Conditions	Cycles	Parameter	BA	PhPA	PhLA	p-HBA	p-HPhAA	p-HPhPA	HVA	p-HPhLA
D2H2	Undiluted serum	15	MNR, %	105 ± 3	57 ± 9	22 ± 4	54 ± 5	53 ± 4	70 ± 10	61 ± 9	30 ± 10
			RSD, %	1	6	8	4	3	7	6	15
	Diluted serum	15	MNR, %	91 ± 1	90 ± 60	40 ± 20	60 ± 20	50 ± 20	70 ± 40	70 ± 30	24 ± 8
			RSD, %	1	27	16	14	14	22	15	14
MN200	Undiluted serum	15	MNR, %	89 ± 4	65 ± 3	19 ± 7	60 ± 20	51 ± 6	70 ± 30	60 ± 10	20 ± 10
			RSD, %	2	2	14	13	5	15	7	28
	Diluted serum	15	MNR, %	89 ± 1	79 ± 8	30 ± 10	70 ± 20	60 ± 20	80 ± 30	74 ± 20	24 ± 6
			RSD, %	1	4	19	11	12	14	11	11
		10	MNR, %	90 ± 3	80 ± 20	30 ± 10	60 ± 10	50 ± 10	67 ± 6	65 ± 9	21 ± 8
			RSD, %	1	13	21	7	9	4	6	15
		7	MNR, %	90.7 ± 0.5	80 ± 20	31 ± 4	70 ± 10	60 ± 10	71 ± 3	70 ± 10	26 ± 6
			RSD, %	1	8	5	8	9	2	7	10
		4	MNR, %	93 ± 7	70 ± 20	27 ± 6	70 ± 30	60 ± 20	70 ± 20	70 ± 30	20 ± 10
			RSD, %	3	10	9	16	16	12	15	20

## Data Availability

The original contributions presented in this study are included in the article/Supplementary Material. Further inquiries can be directed to the corresponding author.

## Supplementary Materials

The following supporting information can be downloaded at https://www.mdpi.com/article/10.3390/ijms27146443/s1.

## Abbreviations

The following abbreviations are used in this manuscript:

BA	Benzoic acid
BSTFA	*N,O*-bis(trimethylsilyl)trifluoroacetamide
D5-BA	Benzoic acid-2,3,4,5,6-d5
3,4-diHBA	3,4-Dihydroxybenzoic acid
GC-MS	Gas chromatography–mass spectrometry
GLMM	Generalized linear mixed model
HCLPS	Hypercrosslinked polystyrene
MEPS	Microextraction by packed sorbent
MNR	Mean normalized recoveries
p-HBA	4-Hydroxybenzoic acid
p-HPhAA	4-Hydroxyphenylacetic acid
p-HPhLA	4-Hydroxyphenyllactic acid
p-HPhPA	4-Hydroxyphenylpropionic acid
PhPA	3-Phenylpropionic acid
PhLA	3-Phenyllactic acid
RAM	Restricted access material/media
RSD	Relative standard deviation
SPE	Solid-phase extraction
