# Supplementary material for: A 3D-Printed Micro-Solid-Phase Extraction Device with Hypercrosslinked Polystyrene Sorbents for Highly Reproducible Aromatic Acid Analysis in Blood Serum
Source: Int J Mol Sci. 2026 Jul 20;27(14):6443. doi: 10.3390/ijms27146443 (PMC13410271; doi:10.3390/ijms27146443)
Supplement: Supplementary file 1 [file ijms-27-06443-s001.zip › ijms-4426953-supplementary/Suppl. Section S1 and Fig. S1-S3.pdf]

Supplementary Section S1. GLMM analysis

For GLMM analysis, relative recovery data were converted to long format and merged with analyte-specific logP values. Sorbent, analyte, and sample_ID were encoded as factors. The number of cycles was transformed as 1/n and shifted to its minimum, dilution was recoded as 1 − dilution to set the diluted condition as reference, and logP was mean-centered. Missing, non-positive, or logP-incomplete observations were excluded.

```
Family: gaussian  ( identity )
Formula: log(rel_rec) ~
~ sorbent + cycles + dil + logP_sc + (1 | analyte) + (1 | sample_id)
Dispersion: ~ sorbent + cycles + dil + logP_sc + sorbent:dil
Data: df_rel_long

    AIC      BIC   logLik  -2*log(L)   df.resid
  -151.3   -110.7     88.6     -177.3        155

Random effects for Conditional model:
 Groups    Name        Variance Std.Dev.
 analyte   (Intercept) 0.148489 0.3853
 sample_id (Intercept) 0.004651 0.0682
 Residual                    NA     NA
Number of obs: 168, groups:  analyte, 8; sample_id, 21

Conditional model:
              Estimate Std. Error z value Pr(>|z|)
(Intercept)    4.08373    0.14503  28.158  < 2e-16 ***
Sorbent MN200  -0.04781    0.04841  -0.988  0.32327
cycles         0.01522    0.30359   0.050  0.96002
dil           -0.13227    0.04706  -2.810  0.00495 **
logP_sc        0.43416    0.23232   1.869  0.06165 .
---
Signif. codes:  0 '***' 0.001 '**' 0.01 '*' 0.05 '.' 0.1 ' ' 1

Dispersion model:
                Estimate Std. Error z value Pr(>|z|)
(Intercept)      -1.644060   0.165056  -9.961  < 2e-16 ***
Sorbent MN200    -0.841200   0.200537  -4.195  2.7e-05 ***
cycles           -0.182940   1.081948  -0.538   0.5905
dil               0.346213   0.158025   2.191   0.0284 *
```

logP_sc   0.005471   0.128938   0.042   0.9662
Sorbent MN200:dil   0.700652   0.288510   2.429   0.0152 *

Signif. codes: 0 ‘***’ 0.001 ‘**’ 0.01 ‘*’ 0.05 ‘.’ 0.1 ‘ ’ 1

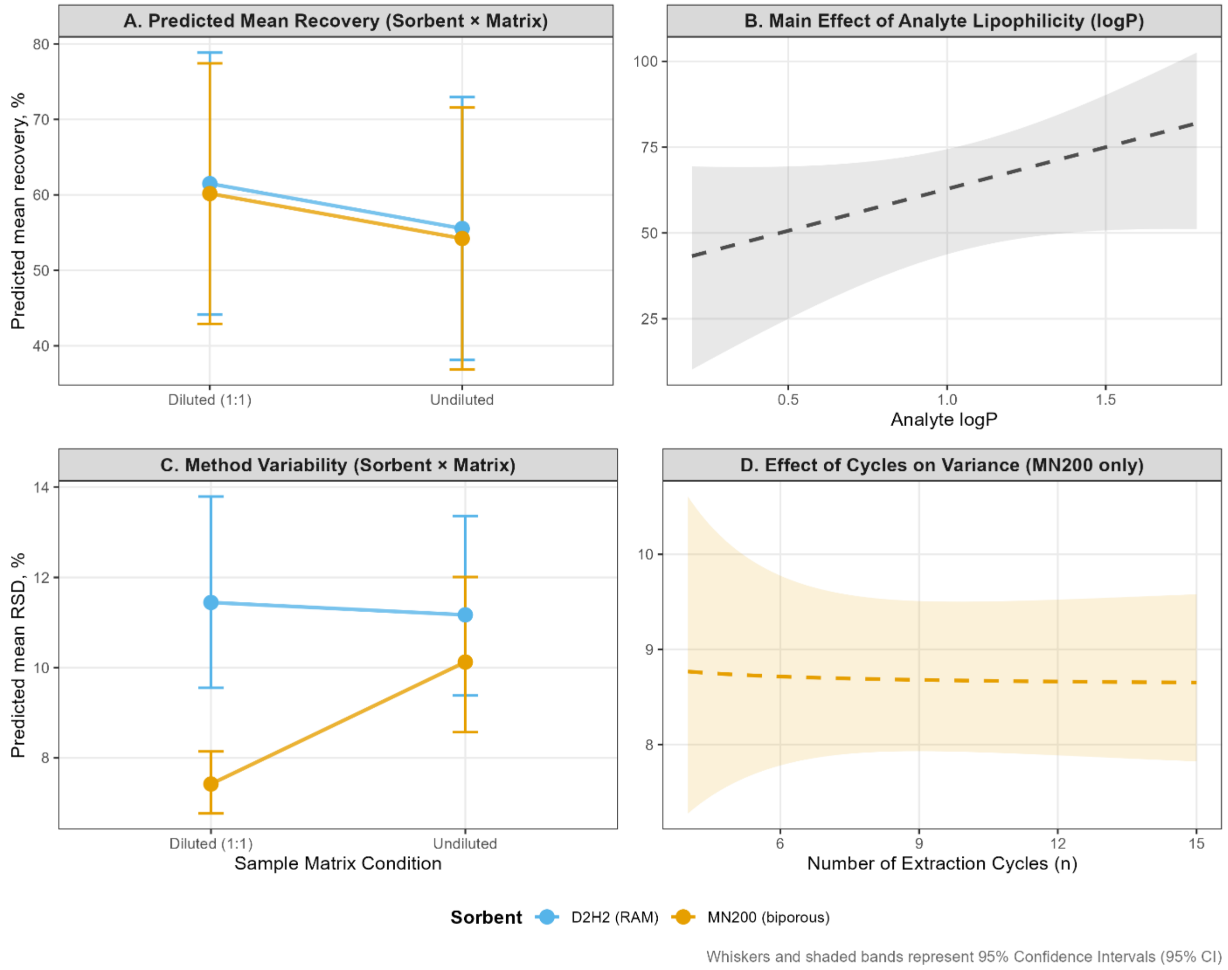

Figure S1. Graphical summary of the GLMM predictions for mean normalized recovery (panels **A**, **B**) and relative standard deviation (RSD) (panels **C**, **D**). In panels **A**, **C**, and **D**, the predicted values are averaged across all analytes to highlight the effects of sample matrix, sorbent type, and the number of extraction cycles. Conversely, in panel **B**, the predicted recovery is averaged across both sorbents and matrix conditions to illustrate the main effect of analyte lipophilicity (logP). Whiskers and shaded bands represent 95% confidence intervals.

Model diagnostics supported the adequacy of the GLMM for estimating the effects of experimental factors on normalized recovery and reproducibility (Figs. S1 and S2). The model converged successfully with a positive-definite Hessian, indicating that the prespecified fixed effects, random intercepts, and dispersion component were estimable from the available dataset of 168 observations across 8 analytes and 21 sample IDs. Multicollinearity among fixed effects was low (VIF range 1.0–1.3). DHARMa diagnostics showed no significant global deviation from the expected residual distribution, overdispersion, or excess outliers (KS test, p = 0.098; dispersion test, p = 0.722; outlier test, p = 1.000; Fig. S1), and Pearson residual plots showed no severe deviation from normality or pronounced residual pattern (Fig. S2). The significant DHARMa quantile test suggested minor residual structure, likely related to unmodelled experimental heterogeneity; nevertheless, the absence of overdispersion, excess outliers, or strong Pearson-residual patterns supported the use of the GLMM for estimating the main experimental effects.

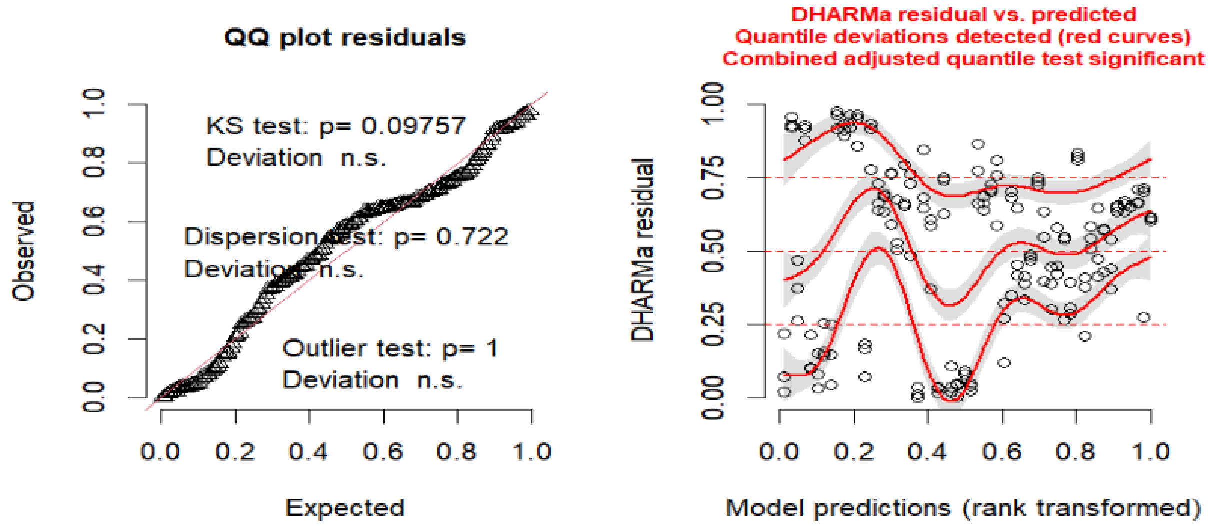

Figure S2. DHARMa residual diagnostics for the GLMM.

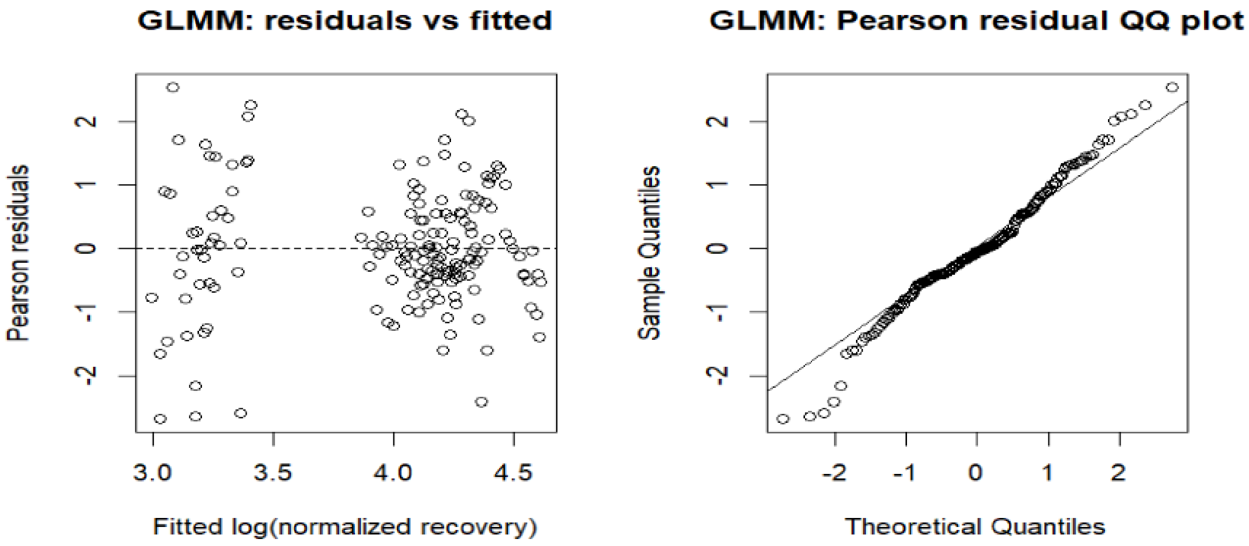

Figure S3. Pearson residual diagnostics for the GLMM.
