# Supplementary material for: A 3D-Printed Micro-Solid-Phase Extraction Device with Hypercrosslinked Polystyrene Sorbents for Highly Reproducible Aromatic Acid Analysis in Blood Serum
Source: Int J Mol Sci. 2026 Jul 20;27(14):6443. doi: 10.3390/ijms27146443 (PMC13410271; doi:10.3390/ijms27146443)
Supplement: Supplementary file 1 [file ijms-27-06443-s001.zip › ijms-4426953-supplementary/Suppl. Table S1 and Fig. S4-S5.pdf]

Supplementary Table S1. Retention times, retention indices, molecular weights of trimethylsilyl derivatives of aromatic acids, and *m*/*z* values with proposed formulas of characteristic ions.

Trimethylsilyl (TMS) derivatives	t_R, min	Retention indices*	*M*, g/mol	*m*/*z*	Probable composition of the ion
2,3,4,5,6-D_5-benzoic acid, *mono-* (surrogate internal standard) $C_6D_5COOSi(CH_3)_3$	9.70	1249	199	110<br>184	$[M\text{-}OSi(CH_3)_3]^+$<br>$[M\text{-}CH_3]^+$
Benzoic acid, *mono-* $C_6H_5COOSi(CH_3)_3$	9.73	1251	194	105<br>179	$[M\text{-}OSi(CH_3)_3]^+$<br>$[M\text{-}CH_3]^+$
Phenylpropionic acid, *mono-* $C_6H_5(CH_2)_2COOSi(CH_3)_3$	12.25	1422	222	104	$[M\text{-}HCOOSi(CH_3)_3]^{+\cdot}$
Phenyllactic acid, *di-* $C_6H_5CH_2CH(OSi(CH_3)_3)COOSi(CH_3)_3$	14.48	1575	310	193	$[M\text{-}COOSi(CH_3)_3]^+$
4-Hydroxybenzoic acid, *di-* $(CH_3)_3SiOC_6H_4COOSi(CH_3)_3$	14.93	1625	282	223<br>267	$[M\text{-}(CH_3)_3\text{-}CH_2]^{+\cdot}$<br>$[M\text{-}CH_3]^+$
4-Hydroxyphenylacetic acid, *di-* $(CH_3)_3SiOC_6H_4CH_2COOSi(CH_3)_3$	15.07	1636	296	179<br>296	$[M\text{-}COOSi(CH_3)_3]^+$<br>$M^{+\cdot}$
4-Hydroxyphenylpropionic acid, *di-* $(CH_3)_3SiOC_6H_4(CH_2)_2COOSi(CH_3)_3$	16.44	1757	310	179<br>192	$[M\text{-}CH_2COOSi(CH_3)_3^+$<br>$[M\text{-}HCOOSi(CH_3)_3]^{+\cdot}$
Homovanillic acid, *di-* $(CH_3)_3SiO(CH_3O)C_6H_4CH_2COO\text{-}Si(CH_3)_3$	16.58	1766	326	179<br>326	$[M\text{-}(CH_2O)COOSi(CH_3)_3]^+$<br>$M^{+\cdot}$
3,4-Dihydroxybenzoic acid, *three-* (surrogate internal standard) $((CH_3)_3SiO)_2C_6H_4COOSi(CH_3)_3$	17.11	1808	370	370	$M^{+\cdot}$
4-Hydroxyphenyllactic acid, *three-* $(CH_3)_3SiOC_6H_5CH_2CH(OSi(CH_3)_3)COO\text{-}Si(CH_3)_3$	17.98	1884	398	179	$[M\text{-}CH(OSi(CH_3)_3)COO\text{-}Si(CH_3)_3]^+$

*The retention indices were calculated under temperature-programmed conditions using the following formula:

$I = 100 \times [\ (t_{R,\,i} - t_{R,\,n}) / (t_{R,\,n+1} - t_{R,\,n}) + n]$,

where $t_{R,\,i}$, $t_{R,\,n}$, and $t_{R,\,n+1}$ - retention times of the analyte and *n*-alkanes eluting immediately before and after the analyte, respectively; *n* is the number of carbon atoms in the alkane eluting before the analyte.

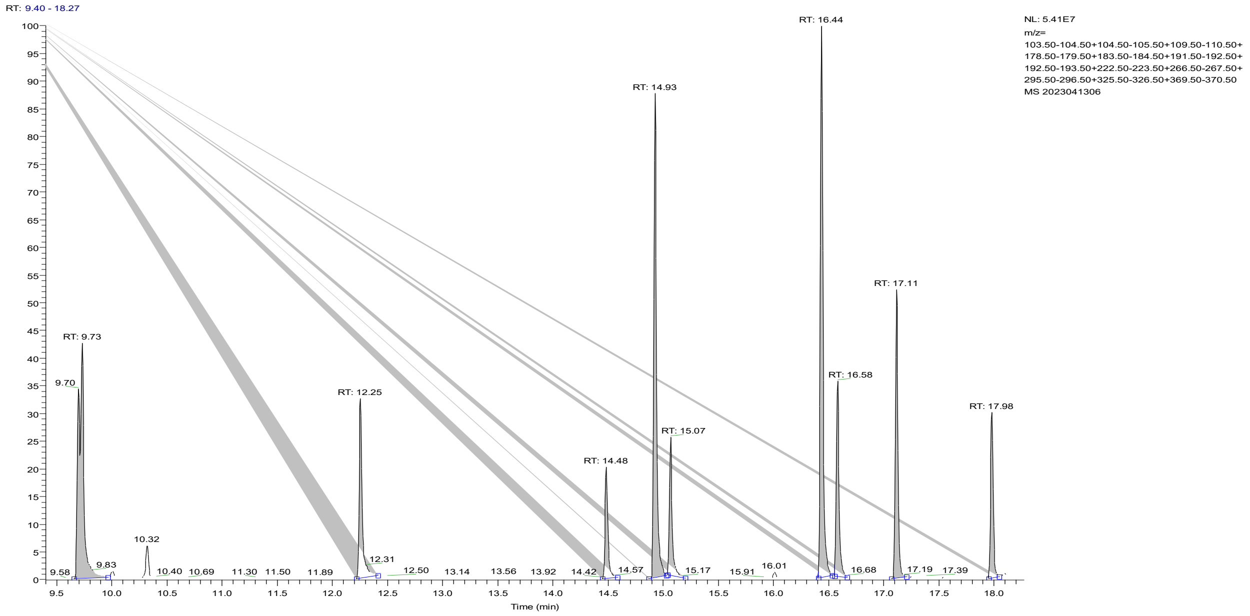

Supplementary Figure S4. A representative chromatogram constructed using all characteristic m/z values (104, 105, 110, 179, 184, 192, 193, 223, 267, 296, 326, 370) was obtained after sample preparation of pooled blood serum from healthy donors supplemented with internal standards and analytes. The correspondence between the retention time and the trimethylsilyl derivative of the internal standards and analytes is presented in Table S1.

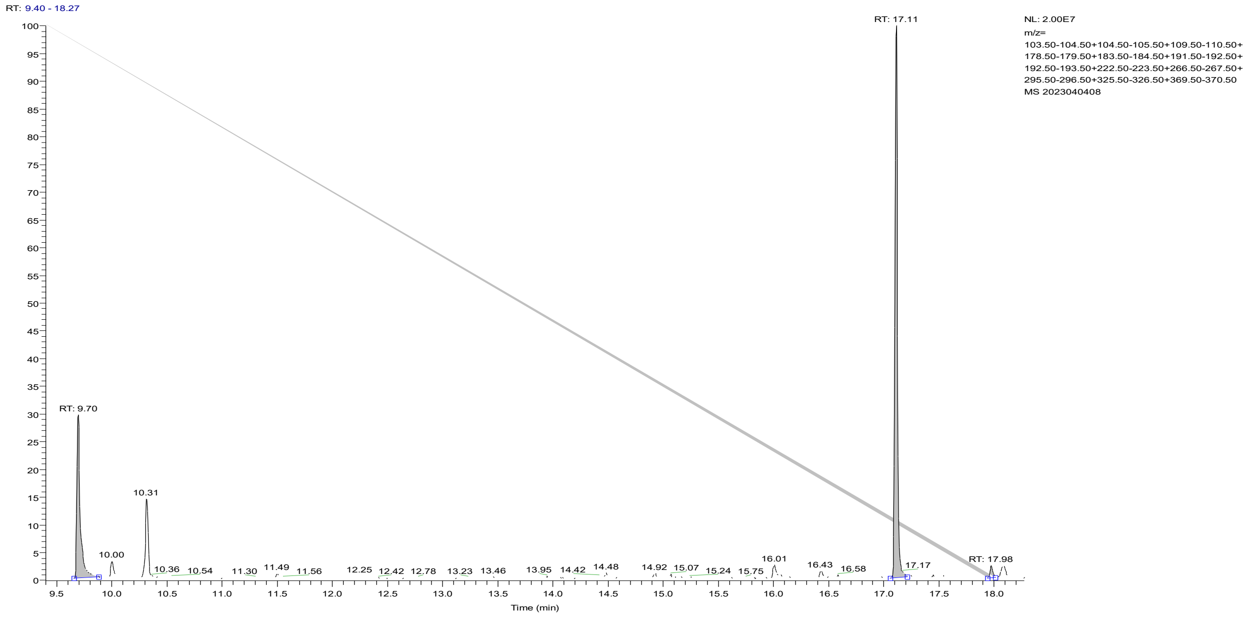

Supplementary Figure S5. A representative chromatogram constructed using all characteristic m/z values (104, 105, 110, 179, 184, 192, 193, 223, 267, 296, 326, 370) was obtained after sample preparation of pooled blood serum from healthy donors supplemented with internal standards. The correspondence between the retention time and the trimethylsilyl derivative of the internal standards and analytes is presented in Table S1.
